# Methylation of Dietary Flavones Increases Their Metabolic Stability and Chemopreventive Effects

**DOI:** 10.3390/ijms10115002

**Published:** 2009-11-18

**Authors:** Thomas Walle

**Affiliations:** Department of Cell and Molecular Pharmacology and Experimental Therapeutics, Medical University of South Carolina, Charleston, SC 29425, USA; E-Mail: wallet@musc.edu; Tel.: +1-843-795-3492

**Keywords:** flavonoids, methylation, methoxyflavones, cancer prevention

## Abstract

Dietary flavones have promising chemoprotective properties, in particular with regard to cancer, but problems with low oral bioavailability and sometimes unacceptable toxicity have made their use as protective additives to normal diets questionable. However, methylation of free phenolic hydroxyl groups leads to derivatives not susceptible to glucuronic acid or sulfate conjugation, resulting in increased metabolic stability. Methylation also leads to greatly improved transport through biological membranes, such as in intestinal absorption, and much increased oral bioavailability. Recent studies also indicate that methylation results in derivatives with increasing potency to kill cancer cells. They also show high potency towards inhibition of hormone-regulating enzymes, e.g., aromatase, important in the causation of breast cancer. Methylation of the flavones may also result in derivatives with diminished toxic side-effects and improved aqueous solubility. In conclusion, it appears that methylation of dietary flavones as well as of other food products may produce derivatives with much improved health effects.

## 1. Introduction

Dietary flavonoids and other polyphenols have long been considered potential chemoprotective agents, mainly in cardiovascular disease and cancer but also in many other disease conditions [1]. This has been supported by numerous cellular studies. This has resulted in an explosion of the market for supplements based on more or, frequently, less scientific evidence. However, in more recent years, in particular in the area of cancer, increasingly in depth research has established these compounds as truly remarkably diverse regarding their multiple cellular mechanisms of chemoprotective action. Whereas initially the flavonoids were purely regarded as antioxidants, it is now clear that this is not the major mechanism of their actions [2]. Instead, a wide range of signaling targets involved in both cancer initiation and promotion have been identified.

The flavonoids have been shown to prevent important carcinogens such as the polyaromatic hydrocarbons from covalently binding to DNA to initiate the carcinogenic process. These effects are mediated by inhibition of the bioactivating enzymes, such as CYP1A1 and CYP1B1 [3–6], and the transcription of these CYP isoforms [6–8]. The chemoprotective function of the flavonoids also includes induction of bioinactivating enzymes such as UDP-glucuronosyltransferase [9], NAD(P)H: quinone oxidoreductase [10] and glutathione *S*-transferase [11].

Extensive studies have focused on effects of dietary flavonoids on cancer cell proliferation and apoptosis. This includes effects on VEGF and HIF-1 expression via PI3K/AKT pathways [12] or via ARNT [13]. Various flavonoids have also been shown to inactivate EGFR [14] and to inhibit thioredoxin reductase [15] and thymidylate synthase [16] as well as to affect cancer cell resistance by targeting the molecular chaperone glucose-regulated protein 78 [17]. Those are all examples of important targets for cancer chemoprevention.

However, when attempting to extrapolate from those cellular studies to the *in vivo* situation, the results are mainly disappointing. Thus, animal studies as well as attempted similar studies in humans have not lived up to the expectations from the *in vitro* studies. The main problem is the very poor oral bioavailability of these flavonoids, mainly due to extensive intestinal and hepatic metabolism.

## 2. Metabolic Stability and Membrane Transport

Most naturally-occurring flavonoids exist as glycosides with various sugars. Numerous studies conclude that these glycosides are not absorbed *per se* but require prior hydrolysis to the aglycones in the intestinal lumen [18–22], which appears to be an efficient process. Our concerns in this review will therefore be focused on the aglycones.

The bioavailability of flavonoids is the cornerstone for extending their *in vitro* biological functions to humans *in vivo* [21,23,24]. Although many flavonoids are absorbed and reach the systemic circulation to some extent, the concentrations in most cases will not be high enough to exert their cellular biological functions *in vivo*. This has been shown in clinical studies of some of the more common dietary flavonoids and other polyphenols, such as chrysin [25], resveratrol [26,27], quercetin [23,28] and the tea flavonoids [29,30]. Taking all the observations in the flavonoid literature together, it appears that extensive conjugation of the free hydroxyl groups is the main reason for the low oral bioavailability of the dietary flavonoids [21,23,24], although transporters may play a role [31–33]. However, whether oxidation also might be important, at least for some flavonoids [3,34,35], is much less clear. Otake *et al.* clearly demonstrated a role for CYP-mediated metabolism of several flavonoids [36,37], first assessing human liver microsomal oxidation, including the CYP isoforms involved, in that commonly used *in vitro* model. Other microsomal studies of CYP-mediated oxidation include the flavonoids biochanin A, prunetin, formononetin, genistein and tangeretin [38,39]. However, isolated microsomes do not reflect the complete metabolic profile, in particular for those flavonoids mainly metabolized by conjugation. Thus, using the human liver homogenate 9,000 g supernatant (S9 fraction) as well as freshly plated human hepatocytes with the flavonoid galangin (3,5,7-trihydroxy-flavone) as the substrate, the role of the CYPs decreased dramatically in comparison with glucuronidation and sulfation. In the S9 fraction, oxidation accounted for only about 2% of total metabolism of galangin [37]. This was very well reflected in the metabolism by the human hepatocytes, the model system that is closest to the *in vivo* situation [37].

**Figure 1.**
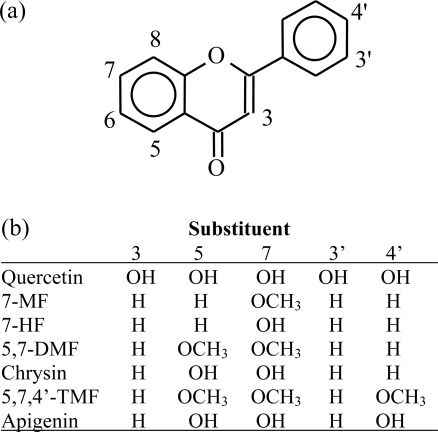
(a) Basic flavone skeleton; (b) structures of some common dietary and other model flavones.

Methylation was examined in our laboratory as a generic approach to cap all free hydroxyl groups in the flavonoids. We focused our interest exclusively on flavones, a flavonoid subclass, see structures in Figure 1.

Polymethoxyflavones such as tangeretin, sinensetin and nobiletin, containing 5–7 methoxy groups, are found in high concentrations in the peel of various Citrus species, whereas the many hydroxylated flavones [40,41] predominate in the juice. The smaller methoxyflavones, with one to three methoxy groups and without any hydroxyl groups, are present in plants that are less utilized for human consumption compared to the polymethoxyflavones. For example, 5,7,4’-TMF, although present in a Citrus species [42], also is present in other plants used in folk medicine [43,44]. 7,4’-DMF has been found in neotropical nutmeg species [45,46]. 5,7-DMF is highly abundant in pepper tree leaves [47]. While none of the small methoxylated flavones are abundant in the common human diet, the mounting evidence of protective properties of these flavones may lead to increased use of their natural sources. Many of these compounds are also available in synthetic form. Parts of this topic have been addressed in previous publications [48–50].

**Figure 2.**
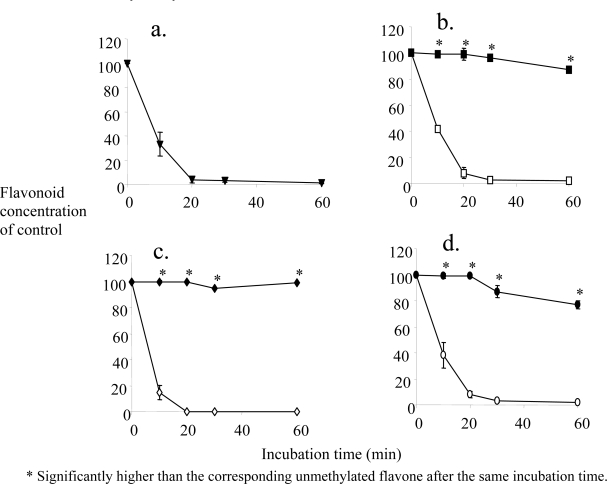
Time-dependent metabolic depletion of unmethylated and methylated flavones in pooled human liver S9 fraction (modified from ref. [51]). (a) quercetin; (b) 7-MF (filled symbols) and 7-HF (open symbols); (c) 5,7-DMF (filled symbols) and chrysin (open symbols); (d) 5,7,4’-TMF (filled symbols) and apigenin (open symbols). Human liver S9 fraction was incubated with UDPGA, PAPS and NADPH and 5 μM flavone and analyzed by HPLC. * Significantly higher than the corresponding unmethylated flavone after the same incubation time.

Our hypothesis was that blocking the free hydroxyl groups of the flavones should eliminate conjugation as the primary metabolic pathway. If the oxidative demethylation rate was slow enough, big improvements in metabolic stability may result. Figure 2 shows the metabolic depletion of unmethylated as well as methylated polyphenols in pooled human liver S9 fractions supplemented with the cofactors for glucuronidation, sulfation and oxidation. Figure 2a shows the metabolic stability for the unmethylated common dietary flavone quercetin, demonstrating rapid disappearance of the parent compound due to extensive metabolism. For quercetin this was due mostly to glucuronidation [51]. This is consistent with its very low oral bioavailability in humans [28]. Similar results were obtained for three unmethylated flavones, 7-hydroxyflavone (7-HF), chrysin (5,7-dihydroxyflavone) and apigenin (5,7,4’-trihydroxyflavone) (open symbols, Figures 2B-D).

For the unmethylated flavones the disappearance from the S9 incubation medium was very rapid, not significantly different from quercetin. For 7-HF sulfation dominated slightly, whereas for chrysin and apigenin glucuronidation was by far the major metabolic pathway. In sharp contrast, the corresponding methylated flavones, 7-methoxyflavone (7-MF), 5,7-dimethoxyflavone (5,7-DMF) and 5,7,4’-trimethoxyflavone (5,7,4’-TMF) (closed symbols), all showed remarkable stability [51].

**Figure 3.**
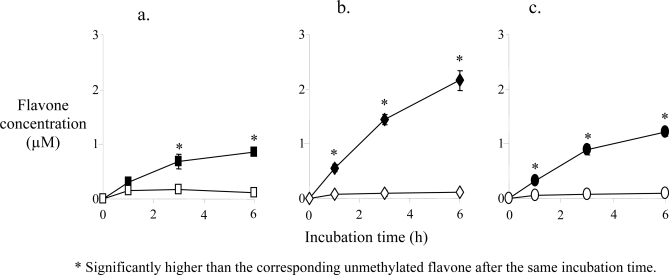
Caco-2 cell transport of methylated versus unmethylated flavones (modified from ref. [51]). (a) 7-MF (filled squares) and 7-HF (open squares); (b) 5,7-DMF (filled diamonds) and chrysin (open diamonds); (c) 5,7,4’-TMF (filled circles) and apigenin (open circles). A 5 μM concentration of the flavones (10 μM for 5,7-DMF and chrysin) in transport buffer was added to the apical chambers of Transwells. Samples were taken from the basolateral side at 0.5, 1, 3 and 6 hr. * Significantly higher than the corresponding unmethylated flavone after the same incubation time.

When examining the absorption of the same three unmethylated versus methylated flavones in Caco-2 cell monolayers, considered the best model of human intestinal absorption [52,53], considerably higher permeability was observed for the methylated compounds (Figure 3). The reason for this higher transport rate cannot be deduced from these experiments, although it most likely is related to the greater metabolic stability of the methylated compounds. The unmethylated flavones were mainly metabolized by sulfation, consistent with the fact that sulfation in the intestine is relatively more important than glucuronidation. Only for chrysin was there a significant contribution from glucuronidation [51].

**Figure 4.**
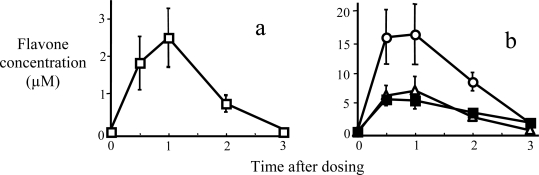
Plasma and tissue levels of 5,7-DMF and chrysin after oral administration of 5 mg/kg in rats. (a) Plasma 5,7-DMF (no chrysin could be detected); (b) tissue 5,7-DMF in liver (○), lung (▪) and kidney (Δ); (mean ± SEM of 5 animals at each time-point) (modified from ref. [54]).

Based on these observations with the hepatic S9 fraction and the Caco-2 cells, it could be predicted that the oral bioavailability of 5,7-DMF would be much greater than that for chrysin. This was tested directly *in vivo* in the rat [54]. 5,7-DMF and chrysin were co-administered by oral gavage at 5 mg/kg, which is a common dose when chrysin is used as a dietary supplement in humans. Only 5,7-DMF was detectable in the plasma, peaking at 2.3 μM at 1 hr (Figure 4a). 5,7-DMF was also easily detectable in liver, lung and kidney tissue at quite high concentrations compared to plasma (Figure 4b). Chrysin was not detectable in any tissue but started to appear in the fecal pellets in the intestinal lumen after 2 hr (not shown).

Thus, it appears that the methylated flavones gain their high metabolic stability as well as high membrane transport properties due to the fact that they are missing a free hydroxyl group, which otherwise can serve as an acceptor for conjugating glucuronic and sulfate groups. The methylated flavones could be *O*-demethylated by cytochrome P450 enzymes and then conjugated as discussed above, although the two conjugation reactions are much preferred to oxidation. To better understand these relationships, we determined the oxidative *O-*demethylation of 15 methoxyflavones using human liver microsomes as the cytochrome P450 source. The oxidation rates, calculated as Cl_int_, varied dramatically, as much as 20-fold (Table 1) [55]. The finding that all four methoxyflavones showing the highest resistance to microsomal oxidation contained a methoxy group in the 5-position indicates that the 5-methoxy group confers special resistance to oxidative metabolism. The number of methoxy groups does not appear to have any effect on the susceptibility to oxidation, as 5-MF, 5,7-DMF and sinensetin with one, two and five methoxy groups, respectively, were quite stable, whereas 7-MF, 7,3’-DMF and tangeretin with the same numbers of methoxy groups were much less stable. The two flavones with a single methoxy substituent in the B-ring, *i.e.*, 3’-MF and 4’-MF, were the methoxyflavones most prone to oxidation. The two tested flavones with both hydroxy and methoxy groups, *i.e.*, kaempferide and especially tectochrysin, were more susceptible to oxidation than most of the fully methylated flavones.

## 3. Anticancer and Hormonal Effects

It is quite clear from the above that methylation of free hydroxyl groups in the flavones results in metabolically more stable derivatives with superior membrane-penetrating properties and thus vastly improved bioavailability. This may also be expected to lead to greater biological effects. This was tested in several cancer *vs.* non-cancer cells.

Central to protective effects by dietary flavonoids at the promotion stage of chemically-induced carcinogenesis is the ability to inhibit cell proliferation. The damage that the carcinogens have inflicted on cellular DNA during the initiation stage is being propagated into a new cell population. This machinery, *i.e.*, clonal expansion, is highly complex, geared towards giving the cells immortality by stimulating mitogenesis and/or decreasing cell death by inhibiting apoptosis. Protective effects at this stage are critically important. This has been demonstrated in cell culture with unmethylated flavonoids and other polyphenols, as discussed briefly in the Introduction, affecting numerous signal transduction pathways.

Some of the polymethoxylated citrus flavonoids have also in preliminary studies demonstrated antiproliferative properties [56,57]. However, the effect of methylation of the flavonoids on their antiproliferative effects has not been clarified. This is in particular true for the smaller methoxylated flavones. Many studies in the past have assumed that the free hydroxyl groups of the flavonoids and other polyphenols are necessary for biological effects. The long held belief that their antioxidant properties were responsible for the antiproliferative effects has now been found not to be true [2].

**Figure 5.**
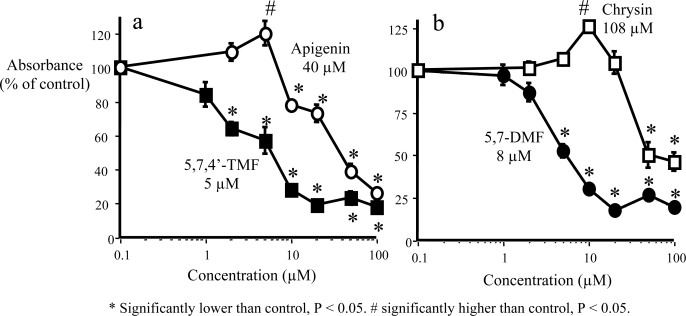
Effect of methylated flavones (a) 5,7,4’-TMF and (b) 5,7-DMF compared to unmethylated analogs apigenin and chrysin, respectively, on SCC-9 cell proliferation (modified from ref. [54]). Cell proliferation, expressed as percent of control (DMSO-treatment), was measured as BrdU incorporation into cellular DNA after a 24-h exposure of the cells to the flavones. Mean values ± SEM (n = 10). The numbers shown in the figure are the calculated IC_50_ values. * Significantly lower than control, P < 0.05. # significantly higher than control, P < 0.05.

The antiproliferative effects of methoxylated versus hydroxylated flavones were directly compared in SCC-9 human oral squamous carcinoma cells. The effects of treatment with 5,7,4’-trimethoxy-flavone (5,7,4’-TMF) versus 5,7,4’-trihydroxyflavone (apigenin), one of the most thoroughly studied unmethylated flavones, are shown in Figure 5a. 5,7,4’-TMF was about eight times more potent than apigenin, with an IC_50_ value of 5 μM. The IC_50_ value of 40 μM for apigenin agrees with most previous studies of this flavone in various human cancer cell lines [58–60]. Very similar results as with 5,7,4’-TMF were obtained for 5,7-dimethoxyflavone (5,7-DMF) compared to its unmethylated analog 5,7-dihydroxyflavone (chrysin) (Figure 5b) [54]. The greater potency of the two methoxylated versus the two hydroxylated flavones could conceivably be due to greater cell uptake of the methoxylated flavones. However, after incubation of SCC-9 cells for up to 24 hr with 25 μM 5,7-DMF or chrysin, the uptake was rapid and virtually identical for the two compounds [54].

A small number of additional methoxylated flavones have been investigated for antiproliferative effects in the SCC-9 cells [54]. The calculated IC_50_ values were 36.5 μM (7-MF), 24.2 μM (7,4’-DMF), and 19.3 μM (tangeretin), respectively. In addition, 5,4’-DMF, 5,3’-DMF and 7,8-DMF showed weaker effects. In MCF-7 human breast cancer cells, the 5-, 7-, and 5,7-methoxyflavanones, *i.e.*, without the C-ring double bond, showed growth inhibitory effects with IC_50_ values around 35 μM, as measured by the MTT assay [61]. In these cells, 5-methoxyflavone was almost as potent as the flavanones and 7-methoxyflavone was more potent than its hydroxyl analog.

In a model of human gastrointestinal malignancies, a very recent study found the anticancer efficacy of a methylated apigenin analog superior to that of apigenin and tricin, an only partially methylated flavone [62]. This finding is very similar to the study in the oral cancer cells above, possibly for the same reasons.

**Figure 6.**
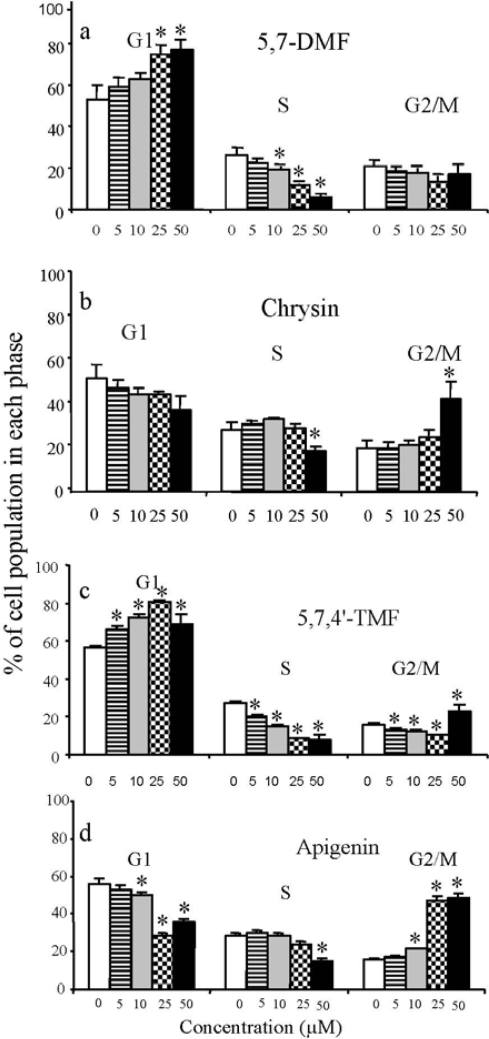
Effect of 5,7-DMF (a) compared to chrysin (b) and 5,7,4’-TMF (c) compared to apigenin (d) on SCC-9 cell cycle progression (modified from ref. [54]). Cells were exposed to varying concentrations of flavones for 48 h. The percentage of cells in G1, S and G2/M phase was measured by flow cytometry after propidium iodide staining. Mean values of 3 experiments with duplicate samples are shown. * Significantly different from control, P < 0.05 or better.

To determine whether the growth inhibitory effect was accompanied by cell cycle arrest, SCC-9 cells were treated with two pairs of flavones for 72 h. These experiments showed that apigenin caused a distinct increase in the G2/M phase population, as has previously been shown [59,60]. In contrast, 5,7,4’-TMF caused a dose-dependent increase in the G1 phase, significant already at 5 μM, with a concomitant decrease in the S phase [54]. Identical results were obtained for 5,7-DMF compared to its unmethylated analog chrysin (Figure 6). The results seen by flow cytometry were thus very similar to those seen in the cell proliferation assay.

To determine if the potent antiproliferative effects observed by the methoxylated flavones on the SCC-9 cells were selective for cancer *vs.* noncancer cells, the effects of 5,7-DMF and chrysin in two additional cancer cell lines were compared with those in two noncancer cell lines. In the FaDu human larynx SCC cells, 5,7-DMF and chrysin showed similar potency as 5,7-DMF in the SCC-9 cells (IC_50_ 8–10 μM). In the MCF-7 human breast cancer cells, both compounds again had similar but slightly lower potency with IC_50_ values of 10–20 μM. In contrast, two normal but transformed human cell lines, *i.e.*, the HET-1A esophageal cells [63] and the BEAS-2B bronchial epithelial cells [64], were much less sensitive to both 5,7-DMF and chrysin with IC_50_ values > 100 μM.

The mechanism of the potent antiproliferative effects of 5,7,4’-TMF and 5,7-DMF compared to their unmethylated analogs has not yet been addressed. However, recent thinking [65,66] suggests that the effect of these methoxyflavones on the AhR-mediated expression, in particular of CYP1A1, may have a direct effect on cell cycle regulation. This is too early to conclude, but may provide a novel set of mechanisms to pursue, connecting cancer initiation with promotion, including the protective effects of the methoxyflavones.

It is however true that many flavonoids have profound effects on both CYP1A1 and CYP1B1 expression, both at the protein and the mRNA levels. These effects appear to be greater for the methylated versus the unmethylated flavones. The effects are cell-specific, as seen for hepatocytes (CYP1A1) [6], lung cells (CYP1A1 and CYP1B1) [8], oral cells (CYP1B1 and CYP1A1) [5] and esophageal cells (CYP1B1) [67].

A specific cancer protective mechanism against hormone-sensitive cancers, which has received much interest lately, is through downregulation of estrogen concentrations. Thus, two methoxylated flavones, 7-methoxyflavone and 7,4’-dimethoxyflavone, have been shown to be potent inhibitors of aromatase, the enzyme responsible for converting testosterone to estradiol (IC_50_ values of 2–9 μM) [68]. Some flavonoids, notably chrysin, have been shown to be potent aromatase inhibitors *in vitro* [69]. However, due to lack of bioavailability [25,51,70], their claims of therapeutic efficacy have never been substantiated. With the methoxylated flavones, this clinical application may be realistic.

## 4. Toxic Side-Effects of Methylated Derivatives

In spite of the evidence of protective effects of flavonoids in many disease states, it is important to realize that toxicity may occur, in particular if ingesting excessive amounts. The latter may sometimes occur after using flavonoids as food supplements. Although being antioxidants, flavonoids can also have prooxidants properties [71], capable of causing oxidative stress, for example through peroxidase-induced phenoxyl radicals [72]. Thus, the flavonol quercetin has been shown to undergo cellular oxidation mediated either by peroxidases [73] or by nonenzymatic chemical reactions and covalent binding to cellular protein and DNA [74,75].

**Figure 7.**
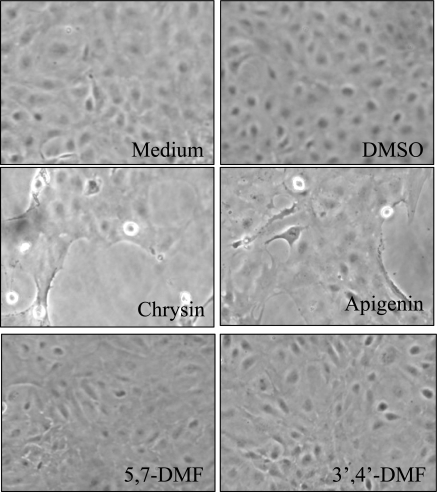
Trout cells after 24-hr exposure to medium, vehicle control, or 25 μM flavones. Dead or dying cells are indicated by arrows (modified from ref. [76]).

To investigate toxic and other effects of xenobiotics in general, cell culture models from a variety of hosts have been used, among which cultured fish cells have found utility in many areas [76]. Rainbow trout hepatocytes have found special utility [77]. This non-cancer cell model was used to examine for toxicity of a few flavones as well as methylated analogs [76]. Growth and health of the trout hepatocytes were monitored by microscopy after treatment with flavonoids or vehicle (DMSO) (Figure 7).

**Figure 8.**
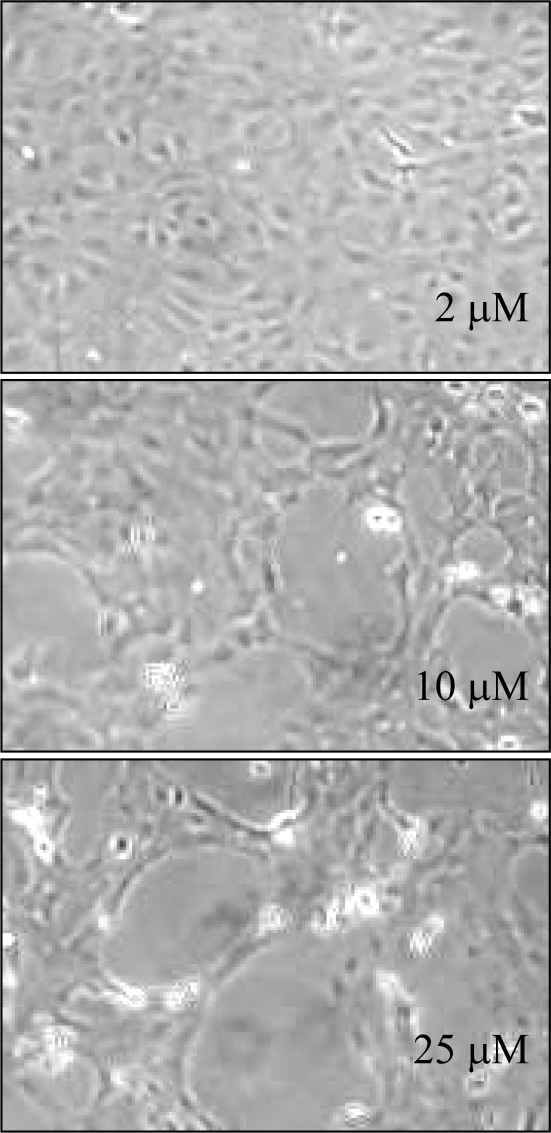
Confluent trout cells treated for 48 hr with various concentrations of chrysin [76].

Treatment with DMSO had no visible effect on growth patterns even up to 48 hr. At 24 hr, all flavonoids caused some inhibition of cell growth, as monitored by confluence levels and protein measurements. Two of the compounds with free hydroxyl groups, *i.e.*, chrysin and apigenin, dramatically reduced cell numbers. Large gaps were seen in the previously semiconfluent monolayers. Additionally, cells were observed lifting off and floating in the medium. In sharp contrast, the methylated chrysin analog 5,7-DMF, showed no toxicity even at a concentration of 25 μM. Another methylated flavone, *i.e.*, 3’,4’-DMF, an aryl hydrocarbon receptor antagonist [78], showed similar lack of toxicity. Chrysin produced a clear concentration-dependent toxicity (Figure 8). A concentration of 2 μM showed no visual adverse effects, whereas 10 μM was clearly toxic and 25 μM even more so.

Trout cell proliferation was measured as BrdU incorporation into newly synthesized DNA of actively proliferating cells. The methoxylated flavone 5,7-DMF showed minimal inhibition with an IC_50_ value of 50–100 μM. This is similar to the response to 5,7-DMF in two normal human cell lines, the lung BEAS-2B cells and the esophageal HET-1A cells [54]. In contrast, chrysin was exceedingly potent, producing an IC_50_ value as low as 2 μM [76].

The mechanism of the observed cell toxicity is not clear from these studies. One possibility is the presence of peroxidases-like activity in these cells, similar to the human myeloid HL-60 cells [76]. Thus, chrysin was oxidized by human myeloperoxidase, but 5,7-DMF was not.

## 5. Solubility of Methylated Derivatives

**Figure 9.**
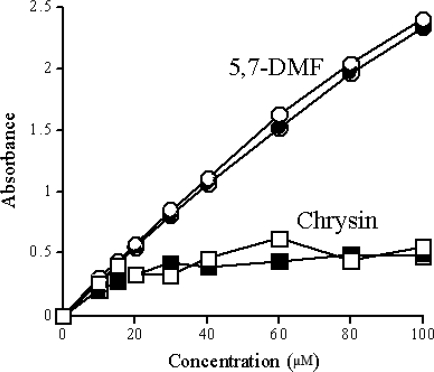
Solubility of 5,7-DMF and chrysin in aqueous solution, *i.e.*, deionized water (open symbols) and Hanks’ buffered salt solution (closed symbols). Measurements were made by UV at 265 nm after removal of insoluble material. Each point is the mean of two determinations [81].

It has long been recognized that the aqueous solubility of flavonoids in general is quite low. This has commonly been observed in *in vitro* experiments. However, *in vivo*, low oral bioavailability has also been attributed to inadequate solubility of the flavonoids in the gastrointestinal tract, in addition to presystemic metabolism. This is certainly true for the extensively studied flavonoid quercetin in animal studies where very large oral doses were used [79], but also in human studies [80]. Low solubility is an obvious problem in general when attempting to design oral dosage regimens. The effect of methylation on the aqueous solubility was therefore examined for 5,7-DMF compared to its unmethylated analog chrysin. The expectation was that the solubility of chrysin would be higher due to its two free hydroxyl groups. The experiments were performed by measuring the UV absorption of the flavonoids after removing undissolved material by centrifugation. Figure 9 shows that 5,7-DMF is soluble in water or pH 7.4 Hanks’ buffer solution up to at least 100 μM. In contrast, chrysin reached maximum solubility at around 20 μM [81]. This surprising finding should be extended to other methylated and corresponding unmethylated flavones. If confirmed, this should favor the use of methylated flavones.

## 6. Conclusions and Future Studies

Methylated flavones comprise a subclass of flavones with distinct properties. In first hand, methylation of the free hydroxyl groups in the flavones dramatically increases their metabolic stability by preventing the formation of glucuronic acid and sulfate conjugates. This also results in increased membrane transport, leading to facilitated absorption and greatly increased bioavailability. Of equally great importance is the finding that methylation results in derivatives with increased intrinsic ability to inhibit cancer cell proliferation, as shown in cancers of the oral cavity [54] as well as in the intestine [62]. The potential therapeutic utility of the methylated flavones has also been advanced by finding that methylation reduces the possibility of toxic side-effects and confers increased solubility. Future studies should be focused on studies demonstrating their clinical effectiveness.

## Figures and Tables

**Table 1.** Elimination half-life and intrinsic clearance of methoxyflavones (5 μM) incubated with human liver microsomes in the presence of NADPH [55].

**Flavone**	**Half-life (min)**	**Cl_int_ (ml min^−1^ kg^−1^)**
5,7-DMF	97.1	12.8
5-MF	68.2	18.3
Sinensetin	44.8	27.8
5,7,4’-TMF	33.3	37.4
3’,4’-DMF	30.9	40.3
7,4’-DMF	28.6	43.6
5,3’-DMF	20.8	60.0
Tangeretin	17.4	71.7
7-MF	15.7	79.4
Kaempferide	15.2	82.0
7,3’-DMF	13.6	91.7
5,4’-DMF	10.5	119.0
3’-MF	8.9	140.0
4’-MF	7.8	161.0
Tectochrysin	4.4	283.0
